# Toxicological evaluation of the ultrasonic extract from *Dichroae radix* in mice and wistar rats

**DOI:** 10.1038/s41598-020-75144-z

**Published:** 2020-10-23

**Authors:** Ling Wang, Zhiting Guo, Dongan Cui, Shahbaz Ul Haq, Wenzhu Guo, Feng Yang, Hang Zhang

**Affiliations:** Key Laboratory of New Animal Drug Project, Gansu Province/Key Laboratory of Veterinary Pharmaceutical Development, Ministry of Agriculture and Rural Affairs/Lanzhou Institute of Husbandry and Pharmaceutical Sciences of Chinese Academy of Agriculture Sciences, 335 Jiangouyan Street, Qilihe District, Lanzhou, 730050 Gansu Province People’s Republic of China

**Keywords:** Drug discovery, Medical research

## Abstract

This study was aimed at evaluating the acute and subchronic toxicity of ultrasonic extract of *Dichroae radix* (UEDR) in mice and rats. High performance liquid chromatography (HPLC) and thin layer chromatogrephy (TLC) were used to detect *β*-dichroine and *α*-dichroine in UEDR for quality control. The levels of *β*-dichroine and *α*-dichroine in UEDR were 1.46 and 1.53 mg/g, respectively. An oral LD_50_ of 2.43 g/kg BW was observed in acute toxicity test. After 28-day repeated oral administration, compared with the control group, treatment-related changes in body weight (BW) and body weight gain (BWG), lymphocyte counts and ratios, as well as in the relative organ weights (ROWs) of liver, kidney, lung, and heart, were detected in the middle- and high-dose groups (*P* < 0.05, *P* < 0.01), no differences were noted in the serum biochemical parameters and necropsy examinations in both sexes at all doses. Histopathological examinations exhibited UEDR-associated signs of toxicity or abnormalities. After 14 days withdrawal, no statistically significant or toxicologically relevant differences were observed in any of the UEDR-treated groups, and the hispathological lesions in the high-dose group were alleviated. Findings showed that long-course and high-dose of UEDR administration was toxic, and showed dose-dependence, the toxic damage was reversible.

## Introduction

Coccidiosis is still one of the most important diseases of chicken, and with extensive use of anticoccidial drugs, resistance has emerged against all introduced drugs ^1–4^. *Dichroae radix*, dry root of *dichroa febrifuga lour.*, known as Chang Shan in Chinese medicine, is used as an antimalarial agent and is officially listed in the Chinese Pharmacopoeia of PR China (Chinese Pharmacopoeia Commission, 2010) and Chinese Veterinary Pharmacopoeia of PR China (Chinese Veterinary Pharmacopoeia Commission, 2015) ^5,6^. Furthermore, *Dichroae radix* has long been used against malarial fever and as an expectorant and antifebrile agent and is a well-known traditional medicine in China ^7^. According to a previous study, *Dichroae radix* has been widely used in Korea as a complementary therapeutic agent to cure unstable fever caused by infection, as well as for the treatment of productive cough in Korea and China ^8^. Some phytochemicals have been extracted and characterized from various effective parts and extracts of *Dichroae radix*, and most of these phytochemicals are plant-derived and effectively bioactive compounds, which can be used as precursors for the synthesis of many drugs with potential for development ^9^. *β*-Dichrorine (febrifugine, dichroine B) and *α*-dichrorine (isofebrifugine, dichroine A), the active components against malaria, were isolated from *Dichroae radix*; *β*-dichroine presented much stronger antimalarial activity compared with α-dichroine, and it works by impairing hemozoin formation, which is required for parasite maturation at the trophozoite stage ^10–13^. Moreover, *β*-dichrorine, a major constituent of *Dichroae radix* has been investigated to minimize parasitemia in *Plasmodium berghei* NK65-infected mice ^14–16^. The results from the immunological tests indicated that an ethanol extract from *Dichroae radix* and the *β*-dichroine contained therein could promote the proliferation of splenic T and B lymphocytes, and had better immune-enhancing activity for macrophage in mice ^17^. Halofuginone hydrobromide, a synthesized analog of *β*-dichroine, has been used to prevent chicken coccidiosis as an antiparasitic feed additive in the poultry industry and is permitted for use by the European Union and the U.S. Food & Drug Administration Agency. However, the use of Halofuginore in clinical applications has been restricted due to the complexity of the synthetic route and the high synthesis cost ^18–20^.

As a plant-derived medicine, *Dichroae radix* has been confirmed to be efficient in the treatment of chicken coccidiosis when utilized as single herb or as the main herb to formulate an herbal complex according to previous studies; however, the contents of active ingredients, such as *β*-dichroine and *α*-dichroine, in coccidiostats prepared with single *Dichroae radix* or *Dichroae radix* complex were always lower. Therefore, a modified ultrasonic-assisted phytomedicine extraction method was selected in this study, and the total alkaloids as active ingredients were extracted from dried roots of *Dichroae radix* to increase the contents of *β*-dichroine and *α*-dichroine therein. The use of *Dichroae radix* and its extractives as anti-coccidial agents is primarily attractive because of their quick effect, lack of associated drug-resistance and accessibility. While considering that *β*-dichroine and *α*-dichroine are alkaloids isolated from *Dichroae radix* and are the active constituents against *E. tenella*, the *β*-dichroine contained therein might possesses adverse side effects in animals and cause some toxicity problems, which might preclude the use of *β*-dichroine as a clinical drug for treating chicken coccidiosis. Additionally, toxicity refers to the level to which a substance can cause harm to a whole organism, as well as substructures of the organism (*i.e.,* an organ such as heart, liver, or kidney). Currently, the use of herbal extracts for the cure of chicken coccidiosis may assure the rising concerns of consumers if it can be confirmed that the extracts are both harmless and valuable. Since the use of plant extracts that contain bioactive compounds can address the growing concern among consumers about drug resistance and safety, it is necessary to assess its safety using standard toxicological methods. Therefore, the evaluation of toxicity for the ultrasonic extract from *Dichroae radix* is necessary to offer scientific statistics for consequent clinical drug security. Regardless of the known facts of the biological actions of *Dichroae radix* the toxicological and consequence profiles have not been sufficiently documented in the application of chicken coccidiosis, and we are not aware of any study in rats simultaneously examining target tissue and sub-acute effects. Thus, this study was conducted to assess the safety of UEDR based on the recommended guidelines and the dose-dependent toxicity in mice and rats. Meanwhile, hematological, serum biochemical and histopathological analyses also aimed to determine the toxicity profile and safe dose. These experiments will provide a basis for the subsequent application of *Dichroae radix* and may assist in the approval of new animal drugs and preparations against chicken coccidiosis.

## Results

### The chemical component analysis of UEDR

**Figure 1:**
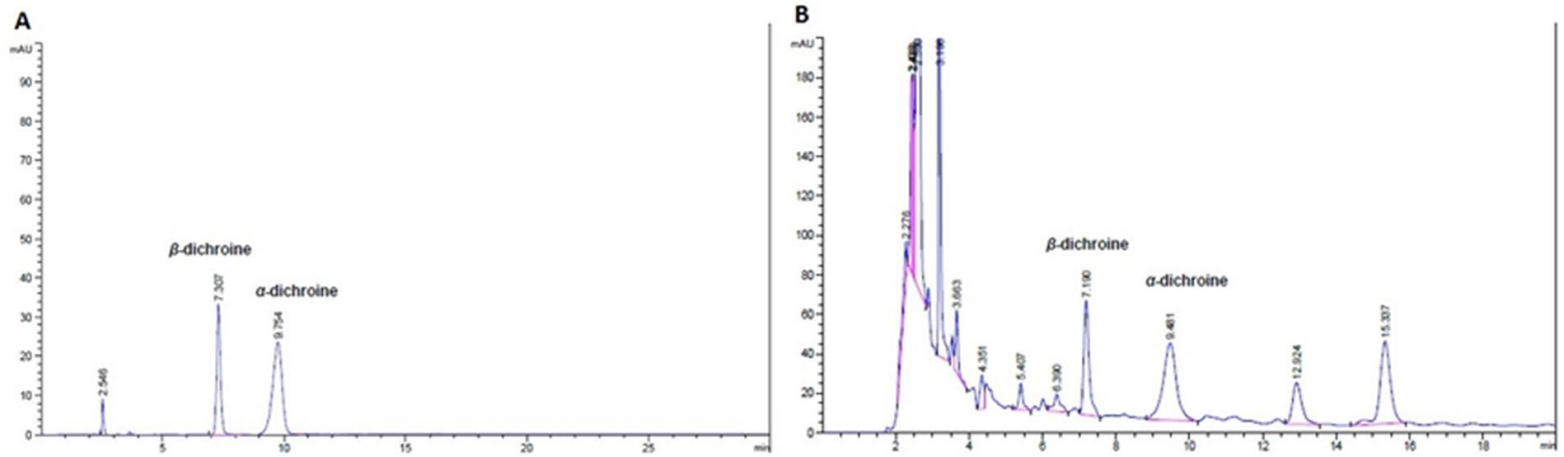
HPLC chromatogram for detecting *β*-dichroine and *α*-dichroine in UEDR. (**A**) *β*-dichroine and *α*-dichroine control, (**B**) the sample of UEDR.

**Figure 2:**
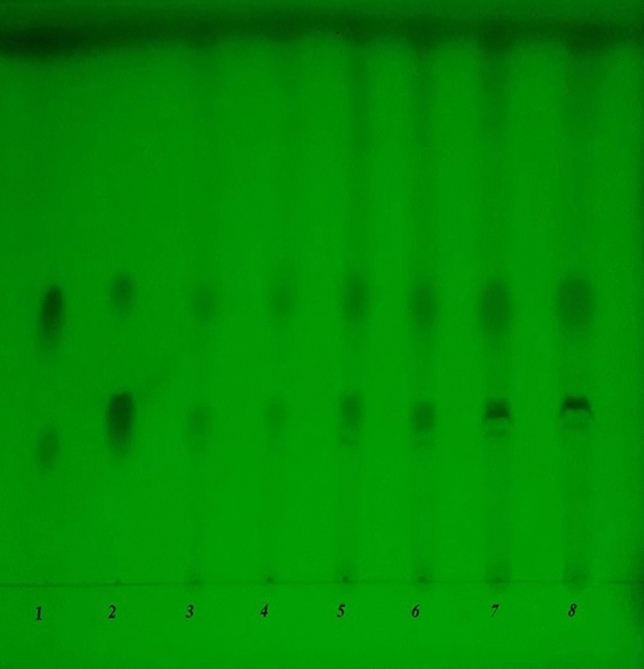
TLC chromatogram for detecting *β*-dichroine and *α*-dichroine in UEDR. (1) *α*-dichroine, (2) *β*-dichroine, (3–8) the samples of UEDR.

*β*-Dichroine and *α*-dichroine, the active compounds of *Dichroae radix*, were presented in UEDR at 1.46 and 1.53 mg/g, respectively, as per HPLC analysis (Fig. 1). According to TLC analysis, *β*-Dichroine and *α*-dichroine, were also presented in UEDR (Fig. 2).

### Acute toxicity studies

Most of the mice orally administered UEDR at a single dose of 3.29, 4.61 g/kg BW exhibited reduced movement following dosing, and a few mice exhibited shortness of breath and neurological symptoms. Prior to death, mice in the high-dose group showed symptoms of body twitching, difficulty in breathing, and piloerection, and death mainly occurred during the observation period of 8–18 h following oral gavage. From the 3rd day following oral administration, the mice that survived returned to a normal behavior state with good appetite, the fur improved and became smooth, and no acute behavioral changes were observed. In addition, the livers and kidneys collected from some of the dead mice in the 3.29 g/kg BW and 4.61 g/kg BW dose groups exhibited congestion and edema via macroscopic examination, while in other organs, no significant gross lesions were detected. Using the Bliss analysis, the LD_50_ was estimated to be 2.43 g/kg BW/oral route, and the 95% confidence interval was 2.03–2.92 g/kg BW. According to the Guidelines of Animal Drug Acute Toxicity Study (the Center for Veterinary Drug Evaluation of Ministry of Agriculture, 2012) ^21^, the UEDR would be considered low toxic in mice.

### Clinical signs and body weight changes in the subchronic toxicity study

No deaths or behavioral changes were observed in rats during the 28-day administration period. However, some rats in high-dose group (0.60 g/kg BW/day) developed symptoms of diarrhea following dosing, lasted for approximately 3 days and then returned to abnormal. Compared with the control group, treatment with UEDR for 28 days caused significant differences in the body weight (BW) and body weight gain (BWG) in male and female rats (*P* < 0.05, *P* < 0.01) between day 21 and day 35, and showed a time- and dose- dependence. Normal BW and BWG was observed in both male and female rats of all the treated and control groups at the end of 14-day withdrawal period (Table 1).

**Table 1:** Effect of UEDR on body weight gain in the subchronic oral toxicity study.

Treatment	Body weight (g, BW)						
	Day 1	Day 7	Day 14	Day 21	Day 28	Day 35	Day 42
**Female ♀**							
Control	102.62 ± 2.83	122.14 ± 3.03	141.41 ± 2.37	161.42 ± 2.58	180.68 ± 3.12	199.37 ± 3.43	223.08 ± 1.44
0.15 g/kg BW	102.51 ± 2.28	121.93 ± 2.54	141.44 ± 1.79	161.23 ± 2.78	179.17 ± 2.77	197.85 ± 3.81	222.73 ± 1.90
0.30 g/kg BW	103.18 ± 4.79	122.47 ± 5.06	141.53 ± 3.28	159.77 ± 3.19	177.78 ± 3.37*	197.95 ± 1.92	221.43 ± 2.66
0.60 g/kg BW	105.19 ± 2.53	123.28 ± 1.99	142.70 ± 1.95	160.65 ± 2.89	176.95 ± 3.39*	193.55 ± 2.34*	220.95 ± 2.26
**Male ♂**							
Control	115.22 ± 2.60	134.39 ± 2.84	154.78 ± 2.36	175.15 ± 2.48	196.60 ± 2.20	216.15 ± 0.59	235.78 ± 0.79
0.15 g/kg BW	115.73 ± 3.05	135.51 ± 2.67	155.43 ± 2.36	175.28 ± 2.07	196.58 ± 2.39	215.70 ± 0.72	234.85 ± 1.09
0.30 g/kg BW	115.66 ± 3.03	134.93 ± 2.70	154.71 ± 2.38	175.10 ± 1.49	195.66 ± 1.82	214.63 ± 1.38	234.65 ± 0.99
0.60 g/kg BW	115.39 ± 1.75	134.80 ± 2.08	154.35 ± 2.25	174.42 ± 2.05	194.50 ± 1.91*	213.53 ± 1.50	234.30 ± 1.53

Treatment	Body weight gain (g, BWG)						
	Week 1	Week 2	Week 3	Week 4	Week 5	Week 6	
**Female ♂**							
Control	19.52 ± 0.70	38.79 ± 2.12	58.80 ± 1.49	78.06 ± 2.88	97.92 ± 3.70	121.63 ± 2.30	
0.15 g/kg BW	19.42 ± 0.50	38.93 ± 1.63	58.72 ± 1.11	76.66 ± 1.56	97.15 ± 3.00	122.01 ± 1.24	
0.30 g/kg BW	19.29 ± 2.11	38.35 ± 2.05	56.59 ± 2.92*	74.60 ± 2.06**	96.70 ± 1.65	120.18 ± 2.70	
0.60 g/kg BW	18.09 ± 1.38	37.51 ± 1.64	55.46 ± 1.85**	71.76 ± 3.08**	90.43 ± 3.64**	117.83 ± 3.11	
**Male ♂**							
Control	19.17 ± 1.16	39.56 ± 0.94	59.94 ± 0.67	81.38 ± 0.60	103.45 ± 0.36	123.08 ± 0.98	
0.15 g/kg BW	19.78 ± 0.97	39.70 ± 1.28	59.55 ± 1.19	80.85 ± 1.00	103.45 ± 1.40	122.45 ± 0.79	
0.30 g/kg BW	19.27 ± 0.66	39.05 ± 1.46	59.44 ± 2.17	80.00 ± 1.38**	102.18 ± 1.01*	122.20 ± 1.52	
0.60 g/kg BW	19.41 ± 1.08	38.96 ± 0.99	59.03 ± 0.73*	79.11 ± 0.63**	99.85 ± 1.34*	120.63 ± 1.14	

Data were presented as means ± SD (n = 10, per group for male and female rats, after 28-day administration; n = 4, per group for male and female rats, after 14-day withdrawal). *P < 0.05, **P < 0.01 *vs.* the control group.

### Relative organ weights (ROWs) in the subchronic toxicity study

After the 28-day oral administration test, the results were obtained for ROWs of liver and kidney (*P* < 0.05, *P* < 0.01), as well as heart and lung (*P* < 0.05), there were significant differences between the high-dose group (0.6 g/kg BW/day) and the control, and female rats exhibited significant changes compared to male rats. The other organs, including spleen, stomach, thymus, ovary, uterus, and testicle, did not present significant differences in any of the UEDR-treated groups compared to the control (*P* > 0.05). Moreover, the ROWs of the treated and control groups did not exhibit abnormal changes after 14 days withdrawal time (*P* > 0.05) (Table 2).

**Table 2:** Effect of UEDR on relative organ weight (ROW) in the subchronic oral toxicity study (× 10^−3^).

Index	28 days treatment (g/kg BW/day)				14 days withdrawal			
	Control	0.15	0.30	0.60	Control	0.15	0.30	0.60
**Female ♀**								
Heart	3.94 ± 0.12	4.06 ± 0.07	4.24 ± 0.38	4.92 ± 0.08*	3.80 ± 0.11	3.42 ± 0.16	3.58 ± 0.19	3.90 ± 0.15
Liver	39.56 ± 0.26	38.36 ± 0.44	38.04 ± 0.51	36.98 ± 0.69**	37.00 ± 0.66	36.22 ± 0.54	37.48 ± 0.51	36.50 ± 0.62
Spleen	2.08 ± 0.12	2.16 ± 0.09	2.16 ± 0.10	2.04 ± 0.13	2.06 ± 0.68	1.96 ± 0.24	2.00 ± 0.04	2.00 ± 0.00
Lung	5.92 ± 0.19	6.22 ± 0.20	6.40 ± 0.14	6.68 ± 0.15*	6.34 ± 0.14	5.96 ± 0.21	6.40 ± 0.11	6.10 ± 0.00
Kidney	6.86 ± 0.17	7.24 ± 0.12	7.56 ± 0.13*	7.64 ± 0.21**	6.82 ± 0.14	6.66 ± 0.23	6.90 ± 0.21	6.43 ± 0.17
Stomach	98.38 ± 0.68	99.12 ± 0.82	100.16 ± 0.44	99.72 ± 0.48	99.34 ± 0.55	99.18 ± 0.53	99.68 ± 0.71	100.03 ± 0.33
Thymus	1.33 ± 0.04	1.39 ± 0.02	1.40 ± 0.02	1.43 ± 0.02	0.97 ± 0.01	0.98 ± 0.01	0.99 ± 0.01	0.98 ± 0.01
Ovary	0.59 ± 0.01	0.60 ± 0.01	0.58 ± 0.02	0.59 ± 0.01	0.54 ± 0.01	0.56 ± 0.01	0.54 ± 0.01	0.53 ± 0.02
Uterus	1.58 ± 0.04	1.58 ± 0.02	1.60 ± 0.02	1.61 ± 0.01	1.42 ± 0.02	1.48 ± 0.01	1.42 ± 0.02	1.44 ± 0.04
**Male ♂**								
Heart	3.46 ± 0.13	3.48 ± 0.13	3.32 ± 0.19	3.38 ± 0.10	3.42 ± 0.08	3.46 ± 0.05	3.28 ± 0.92	3.25 ± 0.06
Liver	38.08 ± 0.31	37.54 ± 0.20	37.40 ± 0.17	37.04 ± 0.26*	35.18 ± 0.97	35.36 ± 1.05	35.98 ± 0.79	37.40 ± 0.15
Spleen	2.28 ± 0.08	2.20 ± 0.22	2.08 ± 0.12	2.10 ± 0.10	1.76 ± 0.07	2.02 ± 0.18	1.96 ± 0.10	2.05 ± 0.13
Lung	4.92 ± 0.24	6.16 ± 0.52	5.76 ± 0.25	6.42 ± 0.58*	5.48 ± 0.14	5.18 ± 0.10	5.32 ± 0.19	5.82 ± 0.48
Kidney	6.16 ± 0.24	6.92 ± 0.24	6.56 ± 0.33	7.18 ± 0.26*	5.90 ± 0.11	5.92 ± 0.08	5.88 ± 0.11	5.92 ± 0.48
Stomach	94.40 ± 2.06	92.54 ± 0.68	91.46 ± 0.79	88.90 ± 1.22	98.06 ± 0.43	96.74 ± 0.70	96.70 ± 0.25	98.15 ± 0.72
Thymus	1.34 ± 0.04	1.40 ± 0.01	1.39 ± 0.01	1.38 ± 0.02	1.01 ± 0.02	1.01 ± 0.01	1.00 ± 0.01	1.00 ± 0.02
Testicle	9.24 ± 0.22	9.34 ± 0.12	9.48 ± 0.07	9.40 ± 0.19	8.70 ± 0.07	8.51 ± 0.07	8.52 ± 0.12	8.62 ± 0.06

Data were presented as means ± SD (n = 6, per group for male and female rats, after 28-day administration; n = 4, per group for male and female rats, after 14-day withdrawal). *P < 0.05, **P < 0.01 *vs.* the control group.

### Hematological and serum biochemical analyses in the subchronic toxicity study

The lymphocytes levels in female rats in high-dose group increased significantly after 28-day repeated oral administration compared with the control rats (*P* < 0.05), and no significant differences in other hematological parameters in female rats in any of the UEDR-treated groups were found. In the male rats, no marked changes were observed in the levels of 11 hematological parameters in any of the dose groups (*P* > 0.05) after continuous dosing for 28 days. Furthermore, there were no abnormal findings in the 11 hematological parameters in male and female rats of all dose groups after 14 days withdrawal time (Table 3).

**Table 3:** Effect of UEDR on hematological indexes in rats in the subchronic oral toxicity study.

Index	28 days treatment (g/kg BW/day)				14 days withdrawal			
	Control	0.15	0.30	0.60	Control	0.15	0.30	0.60
**Female ♀**								
RBC (× 10^12^/L)	7.67 ± 0.17	7.76 ± 0.21	7.85 ± 0.16	7.65 ± 0.20	7.48 ± 0.15	7.48 ± 0.13	7.50 ± 0.31	7.52 ± 0.25
Haemoglobin (g/L)	160.33 ± 3.27	158.83 ± 2.62	157.60 ± 3.44	158.20 ± 3.70	161.00 ± 2.53	160.75 ± 3.41	160.75 ± 3.15	160.50 ± 2.64
Hematocrit (%)	46.13 ± 0.95	46.02 ± 1.22	45.62 ± 0.76	44.64 ± 1.02	47.45 ± 2.19	46.83 ± 2.55	47.88 ± 2.54	48.38 ± 2.15
MCV	60.17 ± 0.45	59.70 ± 0.70	59.10 ± 1.17	59.20 ± 1.31	61.48 ± 1.84	61.60 ± 2.29	60.18 ± 5.72	61.63 ± 2.47
MCH	20.30 ± 0.79	20.03 ± 0.52	20.08 ± 0.21	19.82 ± 0.30	19.75 ± 0.43	19.98 ± 0.23	19.83 ± 0.21	19.68 ± 0.59
MCHC (g/L)	347.33 ± 5.06	345.17 ± 4.50	345.20 ± 2.69	344.40 ± 5.12	323.00 ± 4.73	322.25 ± 7.32	323.75 ± 9.18	324.00 ± 8.37
WBC (× 10^9^/L)	11.20 ± 1.19	11.37 ± 1.43	12.04 ± 0.95	11.88 ± 1.06	11.33 ± 0.78	11.40 ± 1.03	11.45 ± 0.98	11.50 ± 0.98
Lym (× 10^9^/L)	8.35 ± 0.96	8.37 ± 1.45	9.18 ± 1.02	9.90 ± 0.69*	8.36 ± 0.05	8.39 ± 0.17	8.42 ± 0.12	8.55 ± 0.48
Lym (%)	72.63 ± 3.69	73.13 ± 4.10	75.10 ± 4.29	82.48 ± 3.94*	71.68 ± 2.27	71.53 ± 4.15	72.45 ± 1.15	73.65 ± 1.76
Platelet (× 10^9^/L)	1036.50 ± 46.95	1057.00 ± 36.82	1055.80 ± 34.94	1071.20 ± 35.81	1036.25 ± 55.14	1043.75 ± 60.41	1035.00 ± 42.01	1038.50 ± 36.83
MPV	8.22 ± 0.19	8.12 ± 0.17	8.16 ± 0.28	8.36 ± 0.11	8.25 ± 0.28	8.23 ± 0.09	8.28 ± 0.31	8.25 ± 0.17
**Male ♂**								
RBC (× 10^12^/L)	8.13 ± 0.10	8.24 ± 0.22	8.12 ± 0.62	8.34 ± 0.69	8.02 ± 0.30	8.03 ± 0.24	8.06 ± 0.14	8.03 ± 0.36
Haemoglobin (g/L)	162.67 ± 3.05	162.83 ± 5.23	156.50 ± 10.85	158.67 ± 9.42	158.25 ± 2.35	158.75 ± 2.70	160.00 ± 2.73	159.25 ± 2.63
Hematocrit (%)	50.10 ± 0.70	49.43 ± 1.35	47.55 ± 3.79	48.32 ± 4.04	48.40 ± 2.78	48.23 ± 1.93	49.08 ± 2.86	48.67 ± 2.71
MCV	61.60 ± 0.71	61.02 ± 1.12	61.12 ± 1.41	61.48 ± 1.92	64.10 ± 1.56	63.65 ± 1.88	63.75 ± 0.95	63.80 ± 1.50
MCH	19.75 ± 0.26	19.73 ± 0.28	19.47 ± 0.38	19.05 ± 0.53	19.70 ± 0.78	19.93 ± 0.35	19.75 ± 0.56	19.73 ± 0.61
MCHC (g/L)	324.33 ± 5.52	326.17 ± 5.65	327.83 ± 3.49	328.83 ± 9.60	313.75 ± 2.24	312.25 ± 2.01	311.25 ± 4.03	312.50 ± 4.93
WBC (× 10^9^/L)	11.43 ± 1.41	12.15 ± 1.10	12.25 ± 1.20	12.85 ± 1.39	10.90 ± 3.63	10.65 ± 1.59	10.58 ± 0.58	11.00 ± 0.82
Lym (× 10^9^/L)	8.85 ± 0.99	9.08 ± 0.91	9.47 ± 0.96	9.56 ± 0.94	8.90 ± 0.12	8.85 ± 0.88	8.88 ± 0.36	8.98 ± 0.17
Lym (%)	77.50 ± 2.35	74.87 ± 4.02	77.40 ± 4.34	76.80 ± 2.61	75.88 ± 1.93	75.50 ± 3.12	74.88 ± 2.06	76.10 ± 3.20
Platelet (× 10^9^/L)	1119.00 ± 25.45	1105.00 ± 60.09	1104.83 ± 47.73	1123.17 ± 57.51	1028.50 ± 40.18	1023.75 ± 40.26	1017.50 ± 17.51	1011.50 ± 38.83
MPV	8.42 ± 0.25	8.25 ± 0.18	8.43 ± 0.21	8.32 ± 0.30	8.45 ± 0.76	8.43 ± 0.37	8.45 ± 0.02	8.45 ± 0.45

Data were presented as means ± SD (n = 6, per group for male and female rats, after 28-day administration; n = 4, per group for male and female rats, after 14-day withdrawal). *P < 0.05, **P < 0.01 *vs.* the control group. Abbreviations: RBC, red blood cell; MCV, mean corpuscular volume; MCH, mean corpuscular hemoglobin; MCHC, mean corpuscular hemoglobin concentration; WBC, white blood cell; Lym, lymphocyte; MPV, mean platelet volume.

Compared to the control group, lymphocyte counts and lymphocyte (%) in female rats in high-dose group showed significant differences (*P* < 0.05). however, all other biochemical parameters in 3 UEDR-treated groups were slight increased or decreased after 28-day repeated administration, and all the changes in the levels of 6 parameters were within the normal range of the testing laboratory (*P* > 0.05). 14 days after withdrawal, no differences were observed in the levels of 6 biochemical parameters in both sexes at all doses (Table 4).

**Table 4:** Effect of UEDR on Serum biochemical indexes in rats in the sub-chronic oral toxicity study.

Index	28 days treatment (g/kg BW/day)				14 days withdrawal			
	Control	0.15	0.30	0.60	Control	0.15	0.30	0.60
**Female ♀**								
ALT (U/L)	55.00 ± 3.44	51.67 ± 8.60	53.60 ± 4.74	53.80 ± 3.70	56.00 ± 2.19	53.00 ± 7.85	54.00 ± 7.05	52.00 ± 8.48
AST (U/L)	234.17 ± 16.09	229.83 ± 21.86	235.40 ± 13.36	232.20 ± 17.06	235.50 ± 24.27	225.50 ± 20.58	224.50 ± 19.27	229.50 ± 16.36
TP (g/L)	67.70 ± 2.44	67.26 ± 1.33	66.42 ± 1.30	66.38 ± 1.79	68.60 ± 1.49	68.25 ± 1.71	67.93 ± 1.28	69.10 ± 3.23
TBIL (µmol/L)	1.27 ± 0.17	1.28 ± 0.27	1.22 ± 0.18	1.26 ± 0.22	1.23 ± 0.20	1.25 ± 0.05	1.28 ± 0.16	1.25 ± 0.19
BUN (mmol/L)	9.47 ± 1.02	10.07 ± 1.80	9.20 ± 0.49	10.29 ± 1.13	9.83 ± 0.30	9.90 ± 0.29	9.35 ± 1.32	9.60 ± 2.17
Cr (µmol/L)	39.07 ± 5.91	38.45 ± 4.22	35.12 ± 6.62	39.52 ± 8.00	40.50 ± 2.71	37.88 ± 3.02	36.18 ± 8.49	36.58 ± 8.46
**Male ♂**								
ALT (IU/L)	59.50 ± 7.45	58.50 ± 4.89	59.50 ± 3.12	60.50 ± 4.68	56.00 ± 3.65	55.50 ± 3.80	56.00 ± 4.00	55.50 ± 6.24
AST (IU/L)	220.67 ± 37.58	224.67 ± 5.53	220.00 ± 29.46	216.67 ± 30.44	218.50 ± 28.78	214.25 ± 25.03	217.00 ± 33.17	221.50 ± 44.00
TP (g/L)	67.68 ± 1.13	68.58 ± 4.03	68.75 ± 1.74	69.23 ± 1.93	65.50 ± 0.41	65.10 ± 4.20	66.85 ± 2.36	67.08 ± 2.94
TBIL (µmol/L)	1.22 ± 0.18	1.25 ± 0.08	1.23 ± 0.10	1.28 ± 0.10	1.25 ± 0.09	1.24 ± 0.08	1.25 ± 0.07	1.26 ± 0.11
BUN (mmol/L)	10.37 ± 0.51	10.27 ± 0.74	10.24 ± 0.62	10.63 ± 1.15	10.45 ± 0.30	10.71 ± 0.72	10.81 ± 0.87	10.32 ± 0.97
Cr (µmol/L)	37.57 ± 4.07	38.87 ± 4.98	36.27 ± 5.01	36.28 ± 4.29	37.65 ± 3.46	38.55 ± 0.62	38.25 ± 3.30	39.90 ± 7.59

Data were presented as are means ± SD (n = 6, per group for male and female rats, after 28-day administration; n = 4, per group for male and female rats, after 14-day withdrawal). *P < 0.05, **P < 0.01 *vs.* the control group. Abbreviations: ALT, alanine aminotransferase; AST, aspartate aminotransferase; TP, total protein; TBIL, total bilirubin; BUN, blood urea nitrogen; Cr, creatinine.

### Histopathological analyses of the liver, kidney, spleen, lung, and heart

Observations of necropsy of UEDR treated rats were found to be normal, lacking in any apparent pathological abnormalities in vital organs in any of the treated groups after 28 consecutive days administration. However, histopathological examination of the vital organs revealed abnormal pathological lesions in the UEDR-treated rats compared with the control rats, and the alterations exhibited dose-dependence. There were significant differences between the high-, middle-dose groups and control groups in terms of lesions.

**Figure 3:**
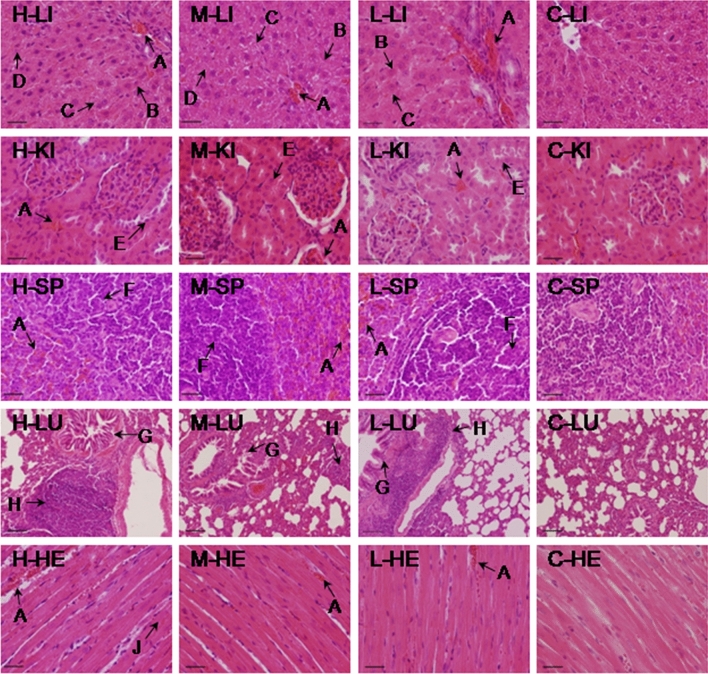
Histopathological analysis of organs in the control (C) and three UEDR-treated groups (high dose, H; middle dose, M; low dose, L) after 28-day administration (H&E stained), scale bar = 50 µm. Livers (LI, 400 ×), kidney (KI, 400 ×), spleen (SP, 400 ×), lung (LU, 100 ×), and heart (HE, 400 ×) (**A**: Congestion; **B**: Granular degeneration; **C**: Vacuolar degeneration; **D**: Necrosis; **E**: Granular degeneration in renal tubular epithelial cells; **F**: Necrosis of splenic lymphocytes; **G**: Bronchial pneumonia; **H**: Interstitial pneumonia; **J**: Myocardial degeneration, slight).

**Figure 4:**
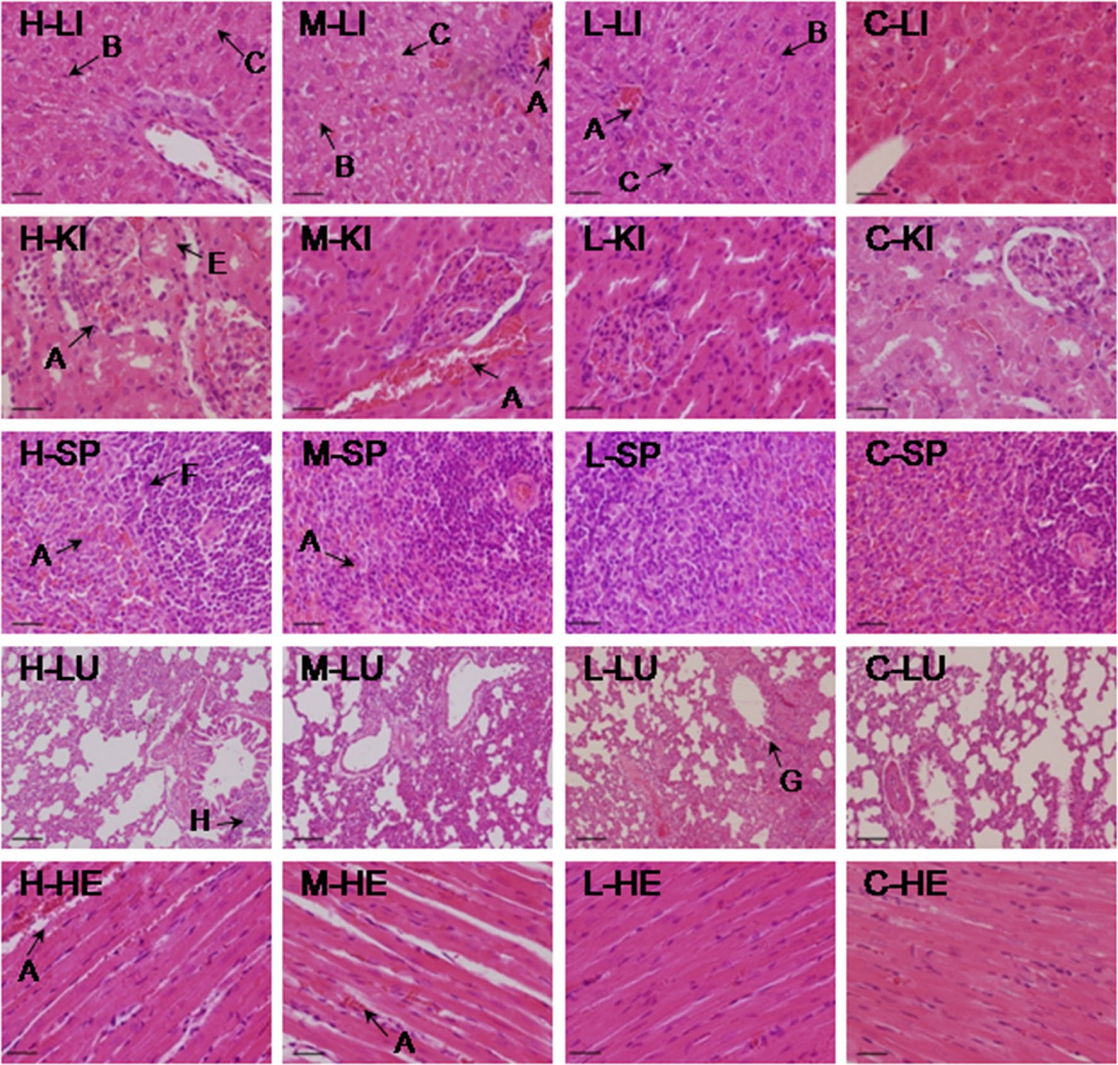
Histopathological analysis of organs in the control (C) and three UEDR-treated groups (high dose, H; middle dose, M; low dose, L) after 14-day withdrawal (H&E stained), scale bar = 50 µm. Livers (LI, 400 ×), kidney (KI, 400 ×), spleen (SP, 400 ×), lung (LU, 100 ×), and heart (HE, 400 ×). (**A**: Congestion; **B**: Granular degeneration; **C**: Vacuolar degeneration; **E**: Granular degeneration in renal tubular epithelial cells; **G**: Bronchial pneumonia, slight; **H**: Interstitial pneumonia, slight).

In the high-, middle-, and low-dose groups, treatment-related histopathological changes were noted in the liver, kidney, spleen, and lung, while in the heart the remarkable pathological alterations were not identified . In the liver, hepatocellular damage was evidence, and presented moderate to severe degrees of necrosis, granular and vacuolar degeneration. In the kidney, moderate to severe granular degeneration in the renal epithelial cells and focal congestion were observed. The spleen exhibited slight to severe degrees of necrosis of splenic lymphocytes and congestion. The histopathological changes in the lungs were characterized by moderate to severe interstitial pneumonia and bronchial pneumonia. Myofibrillar tissue in the heart in the high-dose group presented mild granular degeneration and congestion. These results demonstrated that slight to severe degrees of organ histopathological lesions in UEDR-treated rats existed after 28 days of administration. At the end of the 14-day withdrawal time, no significant hispathological damages were found in the rats in the low- and middle-dose groups, and the pathological lesions in the high-dose group were alleviated. The histological sections of liver, kidney, spleen, lung, and heart of the control and treated rats (day 28 and day 42) are shown in Figs. 3, 4.

## Discussion

*Dichroae radix* exhibits good clinical efficacy in treatments as an antimalarial, expectorant or antifebrile agent in Chinese medicine, and it also has an anticoccidial effect in chickens infected by coccidiosis when used alone or as the main herb to formulate a complex ^22–25^. Therefore, UEDR requires a deeper evaluation of its efficacy and safety using standard toxicological methods due to its anticoccidial demand for reported medicinal use prior to clinical applications. Control of the quality of medicinal materials and preparations with modern analytical tools is very important to ensure their efficacy. In this study, two active compounds (*β*-dichroine and *α*-dichroine) of *Dichroae radix* have been identified and assayed using HPLC and TLC. The results showed that these two active compounds have been presented in UEDR, the contents of *β*-dichroine and *α*-dichroine in UEDR are 1.46 and 1.53 mg/g, respectively. We can make a preliminary conclusion that the quality of UEDR remains good under this condition.

In this study, toxicological protocols have been designed to determine LD_50_ value and further evaluate after continuous or repeated exposure of UEDR. The results of acute toxicity test indicated that UEDR had low toxic in mice, which would help to determine the dosage in animal and provide indexes for potential drug activity, as well as to determine the target organ toxicity and the bioaccumulation potential for UEDR ^26–28^. Then, the subchronic test was performed to further evaluate toxicity, we did not observe any mortality and significant changes in the general behavior in rats during 28-day repeated oral administration period. However, the body weight (BW) and the body weight gain (BWG) of the rats in high- and middle-dose groups had significantly changed compared to control group (*P* < 0.05, *P* < 0.01) from day 21 to day 35. At the end of 14-day drug withdrawl, no significant differences in BW and BWG was observed in both male and female rats in all the treated and control groups (*P* > 0.05). Based on the results of acute/subchronic toxicity test and LD_50_ value, we throught that UEDR is not relatively safe, and long-term administration of high doses of UEDR is likely to be toxic and has an inhibitory effect on the BWG in rats. Similar to the findings of this study, *β*-dichroine showed an LD_50_ of 2.5–3.0 mg/kg in mice after oral administration and had delayed toxic manifestations. *β*-Dichroine was also tested for cytostatic activity, it was active in killing Ehrlich ascites cells in vitro. It is worthwhile mentioning here that in experimental studies, *β*-dichroine showed an antimalarial activity 50–100 times higher than that of quinine, whereas *α*-dichroine was only slightly active ^8,14,22^. Because the high antimalarial activity was accompanied by a high gastrointestinal toxicity in comparison with quinine, a major toxic side effect of *β*-dichroine is its emetic activity, and the emetic effect of febrifugine has been suggested mainly as a reflex action induced by stimulating the afferent vagi and sympathetic nerves of the gastrointestinal tract ^10,12,18,19^. Therefore, the current research mainly focus on the structure of β-dichroine as the leader to synthesize active congeners with lower toxicity.

The present study revealed that the ROWs of all organs in the low- and middle-dose groups in both sexes were not significantly different from the control group (except for the ROW of female rats in middle-dose group). However, the ROWs of the heart (only in female rats), lung, and kidney in the high-dose group increased significantly compared with the control group (*P* < 0.05, *P* < 0.01). In contrast, the ROW of the liver decreased significantly (*P* < 0.05, *P* < 0.01), and the female rats showed more obvious changes than the male rats. Considering the OW and ROW are important indicators to diagnose whether an organ suffers treatment-related injury ^29–31^, the reason might be due to the increasing dose and time of drug administration, the toxic damage caused by UEDR gradually increased, and the body weights of rats in the high- dose group increased slowly, which would result in weight loss. Furthermore, no significant differences were observed on the ROWs between the treated and control groups after 14 days withdrawal (*P* > 0.05). This signified that there is mild toxic effect due to oral administration of UEDR at a daily dose up to 0.30 g/kg BW for 28 consecutive days.

In this study, except for the lymphocyte counts and lymphocyte % in female rats in high-dose group, all other tested hematological and serum biochemical parameters in both sexes were within the normal range at any stage of study, and no significant differences were observed between the control and 3 treatment groups. These findings revealed the production of circulating white/red blood cells and platelets in rats was not significantly affected by UEDR, however, the increased level of lymphocyte were not observed in male rats, suggesting a higher sensitivity of females to the treatment and UEDR might has gender-related effects.

As far as biochemical parameters, there were no marked differences in the levels of ALT, TP, TBIL and BUN between the control and UEDR treated groups in both sexes at all tested doses at day 28 and day 42. ALT, specific to hepatocytes and AST, found in liver, cardiac muscle, and kidney are well-known as markers of cell damage, especially hepatocyte necrosis ^32–34^. TBIL, product of hemoglobin degradation, the increase in serum TBIL is an important indicator and sign of liver damage and cholestasis, and also ralated to increased hemolysis ^35–37^. In our study, the non variation of ALT, AST and TBIL demonstrated the absence of hepatocyte necrosis, and UEDR did not induce myocardial injury and serious liver damage in rats. Furthermore, no significant differences in the levels of BUN and creatinine in male and female rats in any of drug groups were noted compared to the control groups, because BUN and creatinine serve as confirmatory markers for renal dysfunction and failure ^38–40^, the above results revealed that a 28-day continuous administration of UEDR had little negative impact on the kidney function in rats. Hematological and serum biochemical parameters, which were considered as the sensitive indicators of the toxicity of drugs and chemicals, and also give an important index for physiological and pathological status in humans and animals in general ^41,42^. The present study suggests that the macroscopic examinations of the organs in male and female rats in 3 UEDR-treated groups did not produce apparent changes compared with the rats in the control groups, and the necropsy results are in agreement with the hematological and serum biochemical analysis. However, these findings were not further confirmed and supported by the histopathological analysis of the livers, kidneys, spleen, and lung, where UEDR showed toxic effects on the vital organs and abnormal tissue damage was observed frequently in the middle- and high-dose groups.

Under the light microscope (in the middle- and high-dose groups, Day 28), granular degeneration and vacuolar degeneration of hepatocytes was observed in the liver. The liver plate in the hepatic lobules was arranged irregularly, and it can be seen that hepatocytes in the centers of the hepatic lobules (*i.e.*, around the central vein of the hepatic lobules) were swollen, necrotic and disintegrating, and the hepatic sinus was congested (Fig. 3). Renal lesions mainly occurred in renal tubular epithelial cells, the cells exhibited swellingand granular degeneration, and bleeding in interstitial spaces, with mild acute proliferative glomerulitis evident (Fig. 3). Splenic lymphocytes exhibited varying degrees of necrosis and congestion, necrosis and disintegration were found in small numbers of spleen parenchymal cells (*i.e.*, lymphocytes and reticulocytes) (Fig. 3). The lungs presented histopathological indications of interstitial pneumonia and bronchial pneumonia. Tube sleeves around the bronchus and blood vessels formed when the infiltration was obvious, and a small amount of edema fluid was seen in the alveolar space. The alveolar wall epithelial cells showed cubic shape due to swelling, hyperplasia, and metaplasia (Fig. 3). The myocardium exhibited mild granular degeneration, and the structure of the myocardial fiber was intact, with slight swelling or thickening observed, changes in exudation in the interstitium were not significant (Fig. 3). After 14-day withdrawal, no obviously histopathological damages were noted in the rats in the low-dose and middle-dose groups (except for the liver), and the lesions of vital organs in the high-dose group was also alleviated markedly (Fig. 4), indicating that long-term and high-dose oral administration of UEDR can lead to damage to organs (liver, kidney, spleen, and lung), but the toxic damages caused by UEDR is reversible.

## Conclusions

In conclusion, present study reports that repeated (subchronic) doses administration of UEDR is likely to be toxic. This biological assessment of the relationship among dose administered, BWGs, ROWs, and target tissues showed that the main toxic organ targets of UEDR were the liver, kidney, spleen, and lung, which showed dose-dependence. Nevertheless, the toxic damages on the vital organs, BWGs, ROWs, and hematological parameters were significantly alleviated or recovered after 14 days drug withdrawal, indicating that the toxic damage caused by UEDR was reversible. Therefore, based on the results of our analysis the dosage should be set according to the clinically recommended dose to ensure safe dosing. Further studies are necessary for the characterization of the active compounds of UEDR and more extensive biological evaluations.

## Materials and methods

### Plant material and preparation of UEDR

*Dichroae radix* (place of source origin: Sichuan province, China; lot numbers: CN-SC-18009) were purchased from Huanghe Chinese medicine market (Lanzhou, China) in October 2018, and authenticated as the dried root of *dichroa febrifuga l*our., genus Dichroa, family Hydrangeaceae, Angiosperms [MPNS (Medical Plant Name Services) accepted scientific name: Hydrangea febrifuga (Lour.) Y.De Smet & Granados] by Prof. Yun Li, Gansu University of Chinese Medicine, China. A voucher specimen (accession number: GUCM 621,222,130,517,114 LY) was deposited at the Herbarium of the College of Pharmacy.

An ultrasonic extraction method was used to prepare UEDR as followings: the solid–liquid ratio was 1:6 (g/mL, w/v) for *Dichroae radix* powder (200 g) and 2% hydrochloric acid solution, ultrasonic wave power at 80 W, extracted 1 h at 50 °C, with three extraction stages. The supernatants collected from each stage were pooled together, the impurities were removed with a small amount of chloroform, and the pH value was adjusted to 10 with strong ammonia. Each sample was extracted 3 times with chloroform, recovered from chloroform, and the UEDR was concentrated (the final concentration was 2 g/mL, w/v), dried and ground into powder. The concluding concentrations of UEDR administered to mice and rats were 0.04 g/mL (w/v), and stored at 4 °C until use. In this test, two active compounds, including *β*-dichroine and *α*-dichroine from the ultrasonic extract of *Dichroae radix* were chosen to be biomarkers in HPLC and TLC evaluation for the quality control.

### High performance liquid chromatography analysis (HPLC)

The contents of *β*-dichroine and *α*-dichroine in UEDR were determined by HPLC. Quantitative analysis of two components in UEDR was performed on a Agilent apparatus (1290 infinity, two solvent delivery systems, and a Photodiode Array detector, Agilent, USA), SB-C_18_ chromatographic column (4.6 mm × 250 mm, 5 µm, Agilent, USA). The mobile phase consisted of acetonitrile and 0.3% (v/v) glacial acetic acid (30:70), and the pH value adjusted to 5.2–6.2 with triethylamine, filtered through a millipore 0.45 mm filter and degassed prior to use. The injection volume was 10 µL. The flow rate was 1.0 mL/min. The detection wavelength for HPLC analysis was set at 265 nm. The column was maintained at 25 °C. Data collection and quantification were performed with Agilent Open LAB A.02.02 CDS ChemStation (Agilent, USA). The peaks of *β*-dichroine and *α*-dichroine were identified by comparison with chemical standards.

### Thin layer chromatography analysis (TLC)

As the active compounds of *Dichroae radix*, the *β*-dichroine and *α*-dichroine in UEDR were assayed by TLC whichwas based on the standardized experimental protocols of the Veterinary Pharmacopoeia of P. R. China (Chinese Veterinary Pharmacopoeia Commission, 2015) ^6^. Chloroform–methanol-NH_3_·H_2_O (9:1:0.1) was used as the developing solvent to develop *β*-dichroine and *α*-dichroine in the silica gel-GF_254_ plate (Qingdao Haiyang Chemical Reagent Factory, China); *β*-dichroine and *α*-dichroine were used as the standard preparation.

### Animals

Sixty Kunming (KM) mice (5–6 weeks old, 18–22 g, male:female = 1:1) were used for the 7-day acute toxicity test. Eighty Wistar rats (6–8 weeks old, 100–120 g) of both sexes were used for the 28-day subchronic toxicity test. The mice and rats with a clean grade [Certificate No. SCXK (G) 2015–001] were obtained from the Laboratory Animal Center of Lanzhou Veterinary Research Institute (Lanzhou, China). The animals were housed by sex in polycarbonate cages filled with hygiene and sawdust bedding, sustained under standard environmental conditions (23–25 °C) with a relative humidity of 55 ± 10% and 12/12 h light/dark cycle ^21,43^. The cage beddings and water bottle were cleaned on a daily basis during the study. The standard compressed rodent diet (Test clean grade, granule rat feed, Nanjing, China) were provided, and sterilized tap water ad libitum. The experiments were initiated after the animals were acclimatized with a 2-weeks quarantine and adaptation period.

### Ethics statement

All experimental procedures were performed according to the principles of the Center for Veterinary Drug Evaluation (CVDE), Ministry of Agriculture, PR China (2012) ^21^ and was also referred to Organization for Economic Cooperation and Development (OECD) Test Guidelines No. 407 (OECD, 2008) ^44^. All animal experiments were conducted in strict accordance with the National Institutes of Health (NIH, 2002)^45^ Guidelines for the Care and Use of Laboratory Animals. The study was approved by the Ethics Committee of Lanzhou Institute of Husbandry and Pharmaceutical Sciences of the Chinese Academy of Agricultural Sciences (Approval No. LZMY 19-039) (Lanzhou, China).

### Acute oral toxicity test

It was performed using the conventional median lethal dose (LD_50_) method according to the Guidelines of Animal Drug Acute Toxicity Study (the Center for Veterinary Drug Evaluation of Ministry of Agriculture, 2012) ^21^. Before administration, all mice were fasted overnight (12 h) with free access to water. Sixty healthy KM mice (18–22 g) were randomly divided into one control group and 5 treatment groups (n = 10, male: female = 1:1 per group). The UEDR was administered intragastrically to each mouse at 5 single doses of 1.20, 1.68, 2.35, 3.29, and 4.61 g/kg body weight (BW). The changes in genaral behavior and mortality of the mice were observed and recorded, the toxic symptoms and signs (i.e. tremors, convulsions, diarrhea, lethargy, and coma) were monitored for 7 consecutive days, and necropsies for the dead mice were performed during the study period. At the end of the experiment, all mice that survived were euthanized by anesthetic overdose of sodium pentobarbital (150 mg/kg BW intraperitoneal, *i.p.*). The gross necropsy changes in vital organs (liver, lungs, kidneys, heart, spleen, stomach, and intestine) were abserved and recorded. The median lethal dose (LD_50_) was estimated using the Bliss software analysis.

### Subchronic oral toxicity test

#### Observations and measurements

The subchronic oral toxicity of UEDR was conducted according to the Guidelines of Animal Drug 28-Day Repeated Dose Toxicity Study (the Center for Veterinary Drug Evaluation of Ministry of Agriculture, 2012) ^21^. Eighty Wistar rats (100–120 g) were randomized into 3 treatment groups and a control group (n = 20, male: female = 1:1 per group): high-dose group (25% LD_50_, 0.60 g/kg BW/day UEDR for 28 days respectively), middle-dose group (0.30 g/kg BW/day), low-dose group (0.15 g/kg BW/day), and the control group received only distilled water. The daily observation focused on changes in general behavior, skin, eyes, fur, and movement, and attention was paid to observe occurrence of any tremors, convulsions, diarrhea, lethargy, sleep, and coma. The body weight of each rat was measured at the initiation of UEDR administration and weekly thereafter. At the end of the drug administration period, 12 rats randomly selected from each group (6 males and 6 females) were sacrificed for blood hematological, serum biochemical, relative organ weight (ROW) and histopathologiacl analysis. The remaining rats continued to be fed for two weeks after the end of the administration for observation of reversibility, and performed the same tests as above on the 14th day after drug withdrawal.

#### Hematology and serum biochemistry

Twenty-four hours after the last administration, 12 rats randomly selected from each group were anesthetized with sodium pentobarbital (50 mg/kg BW *i.p.*, 6 males and 6 females) after a 8 h overnight fast, and drinking water was available. Blood samples were withdrawn through cardiac puncture into EDTA-containing and nonheparinized tubes for hematological and biochemical parameters, serum specimens were obtained after centrifuging at 3000 × g for 10 min at 4 °C (stored at -20 °C until analysis). Hematological and biochemical parameters were examined by an automatic hematology analyzer (Coulter-JT, Coulter Ltd., USA) and an automatic blood biochemical detector (Olympus AU640, Japan), including red blood cells (RBC), hemoglobin concentration, Hematocrit (%), mean corpuscular volume (MCV), mean corpuscular hemoglobin (MCH), mean corpuscular hemoglobin concentration (MCHC), white blood cells (WBC), lymphocyte (Lym) counts and ratio, platelets, and mean platelet volume(MPV); alanine aminotransferase (ALT), aspartate aminotransferase (AST), total protein (TP), total bilirubin (TBIL), blood urea nitrogen (BUN) and creatinine (Cr).

#### Histopathology

After blood collection, rats were euthanized by anesthetic overdose of sodium pentobarbital (150 mg/kg BW *i.p.*). The selected organs (including liver, kidney, spleen, heart, lung, stomach, thymus, and sex organs) were removed, weighed individually, and dissected to observe macroscopic pathological changes. The selected tissue samples (liver, kidney, spleen, heart, and lung) were then fixed with 10% buffered formalin solution for further histopathological observation. All the tissue samples were dehydrated, paraffin-embedded and sectioned according to standard protocols, and then stained with Hematoxylin and Eosin reagent. The tissue integrity, the presence and characteristics of degeneration, necrosis, infiltration of leucocytes, congestion, extravasations of blood or fibrosis were examined under light microscopy. The images were observed and captured under an optical microscope and image acquisition system (Nikon Model Eclipse E200, Japan). The relative organ weight (ROW) was calculated as organ weight (OW) as a percentage of body weight (BW).

#### Statistics

The data are expressed as the means ± standard deviations, and the statistical significance between means was evaluated by one-way ANOVA, followed by LSD and Dunnett's post hoc test for comparisons between UEDR-treated groups and the control (SPSS 19.0 software, Chicago, Illinois, USA). Differences with *P*-values < 0.05 were considered significant. The male rats and female rats were separately evaluated.

## Data Availability

All data generated or analyzed during the current study are available from the corresponding author on reasonable request.

## Abbreviations

UEDR: Ultrasonic extract of *Dichroae radix*
HPLC: High performance liquid chromatography
TLC: Thin layer chromatography
LD_50_: Median lethal dose
BW: Body weight
BWG: Body weight gain
ROW: Relative organ weight
RBC: Red blood cell
MCV: Mean corpuscular volume
MCH: Mean corpuscular hemoglobin
MCHC: Mean corpuscular hemoglobin concentration
WBC: White blood cell
Lym: Lymphocyte
MPV: Mean platelet volume
ALT: Alanine aminotransferase
AST: Aspartate aminotransferase
TP: Total protein
TBIL: Total bilirubin
BUN: Blood urea nitrogen
Cr: Creatinine
